# Accelerometers Provide Early Genetic Selection Criteria for Jumping Horses

**DOI:** 10.3389/fgene.2020.00448

**Published:** 2020-05-19

**Authors:** Anne Ricard, Bernard Dumont Saint Priest, Sophie Danvy, Eric Barrey

**Affiliations:** ^1^Université Paris-Saclay, INRAE, AgroParisTech, GABI, Jouy-en-Josas, France; ^2^Pole Développement Innovation Recherche, IFCE, Gouffern en Auge, France

**Keywords:** horse, kinematics, jumping competition, heritability, genetic correlation, wearable sensor

## Abstract

The aim of this study was to evaluate the genetic component of the locomotor jumping ability, via a wearable accelerometer sensor, and to estimate the genetic correlation with performance in competition, to introduce such criteria in selection schema. A sample of 1,056 young 3-year-old horses were equipped with a 3-dimensional accelerometer during a free jumping test, in regular breeding shows from 2015 to 2017. Seven variables were extracted from the dorso-ventral acceleration curve for the last three jumps over a double bar obstacle of 1.15 m for the front pole and 1.20 m for the back pole with a 1.20 m spread. Variables were the peaks of forelimbs, hindlimbs, and landing acceleration, the duration between peaks at take-off, the peak of forelimb acceleration and start of jump, jump duration and duration between the beginning of the impact of forelimbs and the peak at landing. During breeding shows, judges scored balance, strength, style, and reactivity for free jumping and jumping tests under saddle. Jumping competition results were recorded by logarithm of the sum of points earned in each competition. All horses in official competitions were included, i.e., 160,257 horses born in 1997 with a total of 649,491 annual performances. An animal mixed model with complete pedigree over four generations (353,236 horses) were used with fixed effects of jumping test location and date, morning/afternoon, gender, month of birth, rank of jump for accelerometric data, effect of year of competition, combined with age and gender for competition results. As a result, jump duration was the most heritable and repeatable for jump variables: *h*^2^ = 0.16 (0.06), *r* = 0.52 (0.02), while accelerations were moderately heritable (*h*^2^ = 0.05–0.09, *r* = 0.39–0.51). Judgement scores were heritable: 0.21 (0.07)−0.33 (0.09) and were highly correlated. Scores during free jumping were genetically correlated to jump duration: 0.71 (0.15)−0.88 (0.16). Both jump duration and judgement scores were genetically correlated to competition performance: 0.59 (0.13) for jump duration, from 0.60 (0.11) to 0.77 (0.12) for scores. Jump duration and judgement scores can be used as early selection criteria. The advantage of the accelerometric measurement is its objectivity and the ease of recording.

## Introduction

Show jumping, consisting of clearing a set of obstacles in a specific order and duration, is one of the most popular equestrian disciplines, with many national and international competitions (Koenen et al., 2004). This sport has a significant economic impact and genetic research is required to improve all the qualities of these sport horses, their welfare, health, and sport life duration. The ability to jump over a high and/or spread obstacle without knocking it down or being disobedient, and to jump successive combinations of obstacles in a minimum time, requires a large set of physiological qualities from the horse-athlete. These include highly competitive behavior, neurosensitive and neurolocomotor coordination, muscular power, and cardiovascular and respiratory capacity. From a genetic point of view, such a complex trait involves many genes and regulations at different epigenetic levels down to post-transcription. Consequently, little scientific data is available on the genetic component of jumping ability. In horses, heritability estimates of jumping competition records are available for different breeds. Depending on the trait measured (in each competition or with annual or life summary) and model used (including rider effect, level of competition effect), heritability ranges from 0.08 to 0.33 (Thorén Hellsten et al., 2006; Ruhlmann et al., 2009). However, very little data about the genetic components of the different physiological systems involved in jumping exercise are known in horses and other species. In horses, a gene wide association study identified three QTLs on chromosomes 1, 4, and 11 which were significantly associated to jumping performance (Brard and Ricard, 2015). Three candidate genes were localized close to the QTL: the ryanodine gene 2 (RYR2) involved in cardiac calcium regulation, actin alpha 1 (ACTA1) one of the structural proteins of the cytoskeleton, and alpha actinin 2 (ACTN2) which plays many roles such as crosslinker and regulator in the cytoskeleton and in particular in the muscle Z-disc (passive elasticity of muscular sarcomere). In humans, the gene actinin 3 (ACTN3) has been associated with the vertical jump explosive power (Domanska-Senderowska et al., 2019). No genetic data of the locomotor and biomechanical aspects of the jumping capacity could be found in the literature for horses. Interestingly, some studies were performed on the biomechanics of jumping in different frog species and showed an evolution according to the species (Reilly et al., 2016). Thus, we assumed that in horses, the genetic component of the jumping ability is also associated to various locomotor characteristics such as the explosive muscular power of the hindlimbs and a fine tuning of limb coordination at the take-off of jumps.

The first French project on jumping kinematic parameters took place in 1984 (Dufosset and Langlois, 1984). Then, Barrey and Galloux (1997) proposed to use a gait analysis device based on accelerometric recordings of the center of gravity of the horse (Barrey et al., 1994) during jumping. Currently, most of the biomechanical studies on jumping use the tracking of anatomical landmarks with high speed cameras (Fercher, 2017). However, this technique is difficult to deploy in a standard breeding show. The efficiency and productivity of the accelerometric device allows recording more than 1,000 related horses in breeding shows. Recording of accelerometric data was managed in a regular breeding show in France over 3 years, in parallel with traditional scoring by judges. This allows for the genetic study of this trait and judgement scores, both of which can be used as selection criteria for jumping horses.

The aim of this study was to evaluate the genetic component of the locomotor jumping ability measured by accelerometry in breeding shows and to estimate the genetic correlation with performances in competitions. This trait will be compared to judgement scores, traditionally used for selection in these breeding shows, to introduce such criteria in selection schema.

## Materials and Methods

### Horses and Jumping Data Collection Conditions

In the breeding program of the Selle Français breed (SF), young horses aged 3 are routinely inspected for their conformation, gait, and jumping ability (free and under saddle) during breeding shows. For 3 years, from 2015 to 2017, the regional breeding shows with the largest number of horses were retained for measurements (four shows in 2015, three in 2016, and three in 2017). The data included 1,056 horses performing 3,125 jumps. The horses were all 3 years old. There were 58% females, 16% geldings, and 25% males. They were all SF except for 28 with only one SF parent, and 12 crossed foreign sport horses.

During regular free jumping tests, all horses were equipped with a gait analysis device (Equimetrix®) fixed with a leather pocket over the caudal part of the sternum at the level of the girth place by means of surcingle (Barrey and Galloux, 1997; Barrey, 2014a). The horses were placed in a circular (Harvrincourt) ring or riding arena and were managed by the same official team. The obstacle combinations included an adjustment obstacle of 60 cm followed by distance for one stride (between 7.0 and 7.4 m long) and then the judged obstacle. The judged obstacle is initially a vertical and then replaced by an oxer. Each horse ended the jumping test with three jumps over a double bar obstacle of 1.15 m for the front pole and 1.20 m for the back pole with a spread of 1.20 m. These last three jumps were used for the jump analysis of the accelerometric data. Simultaneously, a group of judges gave scores on the overall ability of the horses during the free jumping test.

### Jump Analysis by Accelerometry

**Figure 1:**
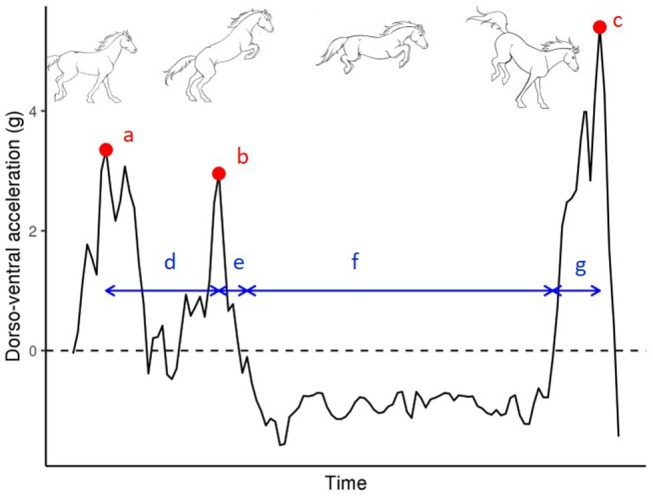
Definition of jump variables used in the analysis from an example of curve of dorso-ventral acceleration during jump with time. (a) the peak of forelimb acceleration at takeoff (FAT), (b) the peak of hind limb acceleration at take-off (HAT), (c) the peak of forelimb acceleration at landing (FAL), (d) the duration between peaks at take-off (DFH), (e) the duration between peak of hindlimb acceleration at take-off and the start of the jump (DHJ), (f) jump duration (DJ), and (g) the duration between the end of the jump and the peak of hindlimb acceleration at landing (DJL).

The body acceleration close to the center of gravity of the horse was recorded with a 3-dimensional accelerometer connected to a microcomputer including large random accessory memory to record the data for up to 7 h (Equimetrix®). All these components are installed in a small aluminum box: 50 × 25 × 110 mm, with a total weight of 240 g. The moment of the jump was analyzed using the jumping tool analysis of the software Equimetrix® on the dorsoventral acceleration visualization and verified with a video tape recording. Jumps with faults were discarded. Raw data included acceleration recorded at 100 Hz per axis in the dorsoventral, longitudinal and lateral axis of the horse from the forelimbs' last impact in the approach stride, to the forelimbs' first impact at landing. Following Barrey and Galloux (1997), only the dorsoventral acceleration was used in further analysis and eight variables were directly extracted from the acceleration curve (Clayton, 1989). These variables are described in Figure 1:

- the peak of forelimb acceleration at takeoff (FAT): maximum of acceleration in the increase of dorsoventral acceleration due to the last forelimbs' support before the jump,
- the peak of hindlimb acceleration at take-off (HAT): maximum of acceleration in the increase of dorsoventral acceleration due to the last hindlimbs' support before the jump,
- the peak of forelimb acceleration at landing (FAL): maximum of acceleration in the increase of dorsoventral acceleration due to the first forelimbs' support after the jump
- The logarithm of the ratio between HAT and FAT.
- the duration between peaks at take-off (DFH): the time spent between the peak of forelimb acceleration and the peak of hindlimb acceleration,
- the duration between peak of hindlimb acceleration at take-off and the start of the jump (DHJ): the time spent between the peak of hindlimb acceleration and the moment when acceleration became negative,
- jump duration (DJ): the time spent with negative acceleration, when the horse is in the airborne phase,
- the duration between the end of the jump and the peak of hindlimb acceleration at landing (DJL): time spent between the beginning of the impact of the forelimbs, i.e., when acceleration became positive, and the peak of acceleration at the landing.

### Judgment Data

During the free jump test, judgements of (1) balance, (2) strength, (3) style, and (4) reactivity were scored on a scale from 1 (poor) to 20 (excellent), but in practice from 10 to 19. Of the horses recorded for accelerometry, 1,045 obtained judgment scores. Similar scores were recorded during the jumping test under saddle. During the jumping test under saddle, the horse performed on a line with combinations of one obstacle up to 0.50 m, followed by 13.5 m, and a second obstacle, an oxer of 0.90 m for the front pole and 1.00 for back pole with a spread of 1.00 m. The entrance was a trotting gait controlled by the ridder.

### Competition Data

To estimate genetic correlation between accelerometric variables and judgment in breeding shows, with the results in an official competition in show jumping, all horses that performed in competitions and were born from 1997 were added to the data. There were 160,257 horses and 649,491 annual performances. Performance in a competition was measured and normalized using the logarithm of the annual sum of points earned in each competition. Points allocated to the horse in a competition depended on the technical difficulty of the event and the rank of the horse. Technical difficulty of the event was assessed by the height of the obstacle, the level of the championship (departmental, regional, national), the category of the rider (amateur/professional), and the table used to score the horse (rules of the table of penalties A or C of the International Equestrian Federation). These technical levels were in accordance with the ranking of horses and riders provided by the French Equestrian Federation (FFE). The rank of the horse was converted into normal scores depending on the rank and the number of participants in the event. The number of points allocated to the horse was the product of the points allocated to the event and the points allocated to the rank (Ricard et al., 2010). The log transformation improved the normality of the distribution.

### Pedigree Data

Pedigree was provided by the Institut Français du Cheval et de l'Equitation (IFCE), who are in charge of zootechnical recording of equids for the count of Stud Book of Selle Français (SBSF). Pedigrees ranging across four generations were added for a genetic analysis. Pedigrees were complete up to the second generation and completeness was 98.3% at the fourth generation. The number of horses in the pedigree data was 353,236 when competition performance is included in the analysis and only 6,900 horses when analyzing only accelerometric data and judgment scores.

The 1,056 horses with accelerometric data were issued from 274 sires (mean progeny per sire 3.9, from 1 to 39; 63 sires had a progeny more than 5). There were 896 mares (maximum size of progeny 4), 386 maternal grand sires (mean grand progeny 2.7; 1–43) and 135 paternal grand sires (mean 7.8 grand progeny; 1–58). The mean inbreeding was 0.014 (from 0 to 0.088, standard deviation 0.015).

### Statistical Model and Quantitative Genetic Analysis

The model was similar for all measurements:

$$\begin{array}{l} y\;=\;Xb+Zu+Zp+e\;, \end{array}$$

with ***y*** being the vector of measurement (either accelerometric data, judgment scores, or competition results), ***b*** being the vector of fixed effects, ***u*** being the vector of random effects of genetic values ($u\sim N\left(0,A\sigma_{u}^{2}\right)$) with ***A*** being the relationship matrix constructed from pedigrees, *p* being the vector of random effects of permanent environmental factors common to the different performances of the same horse $\text{p}\sim N\left(0,I\sigma_{p}^{2}\right)$, and *e* being the vector of residual $\text{e}\sim N\left(0,I\sigma_{e}^{2}\right)$. The model varies according to the fixed effects added:

- For accelerometric data: effect of jumping test location combined with date (10 breeding shows with three being over 2 days, so 13 levels), effect of morning/afternoon (two levels), effect of gender (female vs. male and geldings, two levels), effect of month of birth (four levels: until March, April, May, June, and after June), effect of successive number of jumps (first, second, and third monitored jump, three levels).
- For judgment scores: effect of jumping test location combined with date (10 breeding shows with three being over 2 days, so 13 levels), effect of gender (female vs. male and geldings, two levels), effect of month of birth (four levels).
- For competition: effect of year of competition combined with age (125 levels), effect of gender (female vs. male and geldings, two levels)

Wald F statistics tests for fixed effects were obtained using ASREML software (Gilmour et al., 2014) in a preliminary univariate analysis to determine which fixed effects remained. Then WOMBAT software (Meyer, 2007) was used in a multivariate analysis. Both use REML to estimate variance-covariance matrices. The statistical model analyzed a large number of traits: eight accelerometric variables, eight judgment scores, one competition performance, and a large number of horses with competition records. Therefore, the following analysis was done in several steps (i) as a bi-trait for all correlations with competition performance, (ii) as a multiple trait with heritable traits for accelerometric variables, and (iii) as a multiple trait with heritable traits for accelerometric variables with one judgment score at a time.

## Results

### Distribution of the Variables

The Tables 1, 2 provide the elementary statistics of variables involved in the analysis. All estimated variance-covariance matrices are provided in the Supplementary Material.

**Table 1:** Descriptive statistics of accelerometric variables for all jumps (*n* = 3,125) and judgment scores for all horses (*n* = 1,045).

	**Mean**	**S.D**.	**Min**	**Max**	**Skewness**	**Kurtosis**
**Accelerometric variables**						
Forelimb acceleration peak at take-off: FAT, (g)	3.58	0.90	1.40	6.55	0.38	−0.46
Hindlimb acceleration peak at take-off: HAT, (g)	2.09	0.80	0.26	5.87	0.88	1.22
Forelimb acceleration peak at landing: FAL (g)	4.92	0.58	2.84	6.70	−0.50	−0.17
Log(Ratio HAT/FAT)	−0.78	0.49	−2.81	0.73	−0.32	0.24
Duration between peaks at take-off: DFH (s)	0.28	0.06	0.07	0.54	−0.18	0.19
Duration btw hindlimb peak and jump: DHJ (s)	0.07	0.05	0.00	0.39	2.03	5.85
Jump Duration: DJ (s)	0.60	0.06	0.30	0.84	−0.30	0.65
Duration btw jump and peak at landing: DJL (s)	0.08	0.02	0.04	0.21	1.34	4.24
**Judgement scores**						
Free jumping balance (/20)	14.5	1.3	11.0	19.0	0.19	0.19
Free jumping strength (/20)	14.6	1.4	11.0	19.0	0.13	−0.03
Free jumping style (/20)	14.4	1.3	11.0	19.0	0.23	0.09
Free jumping reactivity (/20)	14.7	1.5	10.0	19.0	0.12	−0.13
Jumping test under saddle balance (/20)	14.7	1.2	12.0	19.0	0.03	−0.12
Jumping test under saddle strength (/20)	14.8	1.3	10.0	18.0	0.06	0.12
Jumping test under saddle style (/20)	14.7	1.3	10.0	19.0	0.14	0.27
Jumping test under saddle reactivity (/20)	14.7	1.3	10.0	19.0	−0.13	0.22

**Table 2:** Descriptive statistics of competition data: number of years of performance and statistics on mean of annual criteria.

	**Horses**	**Years/horse**	**Performances**			
	***N***	***n***	**Mean**	**S.D**.	**Min**	**Max**
Horses with jumping analysis data	818	1.78	1.36	1.49	−4.66	4.48
Horses born from 2012	16,853	1.64	0.41	1.86	−5.25	4.96
Horses born before 2012	143,165	4.34	−0.21	1.79	−5.86	7.74

### Effect of Environmental Factors on Jumping Accelerometric Data

The effect of location was always significant over all measurements. Influence of type of ground surface, environmental conditions (noise, horse being excited, and disturbed) were certainly involved in this effect. This result means that a sufficient number of horses must be recorded in each jumping test location to estimate this effect. Other effects were not always significant depending on each measurement. The main influence was successive number of jumps and sex. Month of birth and effect of morning/afternoon were significant only twice on duration variables.

### Heritability, Repeatability, Genetic, and Phenotypic Correlation Between Jumping Accelerometric Variables

First, heritability (h^2^) and (r) repeatability between jumps were estimated using univariate analysis (Table 3). In order to work out the confidence obtained from this kind of data, to estimate breeding values, expected reliability of estimated breeding values (EBV) from three jumps was computed from heritability and repeatability ($reliability\;=\;\frac{3h^{2}}{1+\;2r}$).

**Table 3:** Heritability, repeatability, and reliability of breeding values after three jumps of accelerometric variables (standard error in parentheses).

	**Heritability**	**Repeatability**	**Reliability EBV (three jumps)**
Forelimb acceleration peak at take-off: FAT	0.09 (0.05)	0.39 (0.02)	0.15
Hindlimb acceleration peak at take-off: HAT	0.09 (0.05)	0.42 (0.02)	0.15
Forelimb acceleration peak at landing: FAL	0.05 (0.04)	0.51 (0.02)	0.07
Log(Ratio HAT/FAT)	0.09 (0.05)	0.37 (0.02)	0.16
Duration between peaks at take-off: DFH	0.03 (0.03)	0.24 (0.02)	0.05
Duration btw hindlimb peak and jump: DHJ	0.01 (0.02)	0.21 (0.02)	0.02
Jump Duration: DJ	0.16 (0.06)	0.52 (0.02)	0.23
Duration btw jump and peak at landing: DJL	0.04 (0.03)	0.27 (0.02)	0.07

The peaks of acceleration at take-off (FAT, HAT) were significantly heritable and repeatable among the three jumps. The ratio between hindlimb and forelimb peaks performed similarly. The jump duration (DJ) was the highest significantly heritable (*h*^2^ = 0.16, standard error 0.06) and repeatable variable (0.52, standard error 0.02). The other duration variables had very low heritability (not significantly different from 0) and low repeatability (0.21–0.27).

Phenotypic and genetic correlations were computed only for sufficiently heritable variables (*h*^2^ ≥ 0.05; Table 4). Phenotypic and genetic relationship between variables reveals a slightly different pattern. Phenotypic correlations were low. Following the logic jump order, FAT was rather independent from HAT, HAT was moderately linked to DJ, and DJ was moderately linked to FAL. The ratio HAT/FAT was mostly driven by HAT. Only three genetic correlations were significantly different from 0. First, the obvious relationship between the ratio HAT/FAT and HAT or FAT was similar to phenotypic correlation (highly positively correlated to HAT and negatively to FAT). Second, there was a high genetic correlation between FAT and FAL.

**Table 4:** Phenotypic (below diagonal) and Genetic correlations (above diagonal) between accelerometric variables (standard error in parentheses).

	**1**	**2**	**3**	**4**	**5**
1. Forelimb acceleration peak at take-off: FAT		−0.22 (0.35)	0.63 (0.38)	−0.72 (0.20)	−0.08 (0.30)
2. Hindlimb acceleration peak at take-off: HAT	−0.06 (0.02)		−0.22 (0.46)	0.83 (0.11)	−0.15 (0.29)
3. Forelimb acceleration peak at landing: FAL	0.23 (0.02)	0.12 (0.02)		−0.56 (0.43)	0.38 (0.37)
4. Log (Ratio HAT/FAT)	−0.58 (0.01)	0.81 (0.01)	−0.02 (0.02)		−0.07 (0.29)
5 Jump duration (DJ)	−0.04 (0.02)	0.17 (0.02)	0.18 (0.02)	0.19 (0.02)	

### Genetic Correlation Between Jumping Accelerometric Variables and Competition Performances

All genetic correlations (Table 5) were positive but only one, between performance in competition and jump duration (DJ), was significantly different from 0 and high (0.59). The other high genetic correlation (0.64) between performance in competition and FAL had a high standard error due to the low heritability of this trait (0.05), and was therefore not reliable. For phenotypic forecast, the only variable with significant correlation was also jump duration (DJ) but with a rather low correlation (0.11).

**Table 5:** Genetic correlation between competition performance and accelerometric variables (standard error in parentheses).

	**Genetic correlation**	**Phenotypic correlation**
Forelimb acceleration peak at take-off: FAT	0.17 (0.15)	0.02 (0.03)
Hindlimb acceleration peak at take-off: HAT	0.20 (0.16)	0.01 (0.03)
Forelimb acceleration peak at landing: FAL	0.64 (0.33)	0.02 (0.03)
Log (Ratio HAT/FAT)	0.03 (0.15)	0.00 (0.03)
Jump Duration: DJ	0.59 (0.13)	0.11 (0.03)

### Genetic Correlation Between Judgment Scores

Genetic correlations between the four different scores for free jumping were close to 1. Similarly, the correlation between the four scores for the jumping test under saddle were also close to 1. In both cases, these traits cannot be introduced simultaneously in a multivariate analysis. Phenotypic correlations were also very high (from 0.75 to 0.82). Genetic correlations between scores for the same criteria between the free jumping test and jumping test under saddle were very high: from 0.70 (standard error 0.18) for balance to 0.96 (standard error 0.14) for strength. In contrast, phenotypic correlations were moderate: from 0.25 for balance (standard error 0.03) to 0.31 for strength (standard error 0.03). This can be explained by different teams of judges involved in the two notations, however, the same genetic aptitude was kept.

### Genetic Correlation Between Jumping Accelerometric Variables and Judgment Scores

Genetic correlation between judgement scores and accelerometric data are presented in Table 6. The only genetic correlation significantly different from 0 was between scores and jump duration (DJ), whatever the detailed score: balance, strength, style, or reactivity. These correlations were high:>0.70 for free jumping and around 0.40 for jumping test under saddle.

**Table 6:** Genetic correlations between judgement scores and accelerometric variables (standard error in parentheses).

	FAT^*^	FAL^*^	Log(HAT/FAT)^*^	DJ^*^
**Free jumping**				
Balance	0.26 (0.27)	0.10 (0.35)	−0.11 (0.27)	0.78 (0.15)
Strength	0.19 (0.26)	0.24 (0.35)	0.19(0.25)	0.71 (0.15)
Style	0.12 (0.32)	0.34 (0.38)	0.02 (0.31)	0.88 (0.16)
Reactivity	0.04 (0.31)	−0.04 (0.39)	0.11 (0.29)	0.77 (0.17)
**Jumping test under saddle**				
Balance	0.28 (0.29)	0.36 (0.36)	−0.10 (0.29)	0.41 (0.23)
Strength	0.21 (0.29)	0.20 (0.36)	0.00 (0.28)	0.37 (0.23)
Style	0.21 (0.26)	0.33 (0.31)	0.10 (0.26)	0.39 (0.21)
Reactivity	0.36 (0.28)	0.10 (0.37)	−0.18 (0.28)	0.26 (0.24)

* *FAT, Forelimb acceleration peak at take-off; FAL, Forelimb acceleration peak at landing; HAT, Hindlimb acceleration peak at take-off; DJ, Jump Duration*.

### Genetic Correlation Between Judgment and Performance in Competition

For free jumping, genetic correlations between judgement scores and performances in competition (Table 7) were rather high (0.60–0.77) and significantly different from 0. These correlations were higher than the one found between DJ and performance in a competition. For the jumping test under saddle, genetic correlations between scores and performance in competition were still high but slightly less than for the free jumping test (0.49–0.63). The corresponding phenotypic correlations were moderate (0.14–0.20), and slightly higher in the jumping test under saddle than the free jumping test.

**Table 7:** Genetic correlation between judgment scores and competition performance (standard error in parentheses).

	**Heritability**	**Genetic correlation**	**Phenotypic correlation**
**Free jumping**			
Balance	0.28 (0.08)	0.60 (0.11)	0.14 (0.03)
Strength	0.33 (0.08)	0.72 (0.10)	0.18 (0.03)
Style	0.21 (0.07)	0.77 (0.12)	0.16 (0.03)
Reactivity	0.23 (0.07)	0.67 (0.12)	0.14 (0.03)
**Jumping test under saddle**			
Balance	0.23 (0.08)	0.53 (0.13)	0.17 (0.03)
Strength	0.28 (0.08)	0.63 (0.12)	0.18 (0.03)
Style	0.33 (0.09)	0.56 (0.11)	0.18 (0.03)
Reactivity	0.28 (0.09)	0.49 (0.11)	0.17 (0.03)

## Discussion

Most literature, which aims to study jumping technique, is focused on the description of the movement rather than using it as a tool for selection purposes. In most studies analyzing jumping, authors managed small groups of animals with sophisticated technology. Most often kinematics were studied using high-speed cameras, and sometimes, inertial measurements or force plates. Results of these biomechanical studies describe limb coordination and the horse's style in clearing different types of obstacles. Fercher (2017) published a large review of these studies on jumping with riders. For free jumping, the only technique used was high speed cameras, except for one publication reporting the use of inertial measurement (Barrey, 2014b). Measurements issued from high-speed cameras were either related to the jump (position of the horse to the obstacle) or to the horse (conformation of the horse during jumping). They all focused only on vertical and horizontal axis; no data were found on movement on the lateral axis. We focused the discussion on results in kinematics from literature that can be related to measurements obtained with accelerometric data. Indeed, studies of movement from video camera can derive acceleration values from the derivation of position landmarks. In addition, reciprocally, accelerometric data can provide variables such as jump duration, which is mechanically related to the distance measured by video: height of the center of gravity or length of the jump for example.

### Repeatability of Measurements Over Free Jumps

Studies, which report repeatability of kinematic measurements over jumps in free jumping for the same horse, agree with our results. For free jumping, de Godoi et al. (2014) calculated repeatability of kinematics traits for 108 Brazilian sport horses performing five jumps. Traits considered in this study were jump performance traits: measurements of stride and distance from obstacle, and horse jump related traits: angles of joints. Repeatability was estimated between jumps in the same session, between tests separated by 7 months and between 2 and 3 years of age. Repeatability in the same session was rather high, mostly between 0.40 and 0.60 for jump performance traits and sometimes higher for horse conformation traits up to 0.80. Repeatability over month was also high: about 0.40 and 0.50. They linked these measurements to the probability of successful jumps in the same test (de Godoi et al., 2016). Logistic regression between kinematic traits and probability of successful jumps were not significant except for the last stride distance and take-off distance. In Poland, repeatability was measured for kinematics during free jumping and jumping tests under saddle of official performance tests of up to 141 warmblood stallions (Lewczuk et al., 2006; Lewczuk and Ducro, 2012; Lewczuk, 2017). They found high repeatability in most of the traits. The repeatability for height of selected points of the horse body above the obstacle and liftings of the legs above the obstacle was between 0.50 and 0.60. Repeatabilities were higher for higher obstacles, longer trained, and best horses.

### Ability of Early Measurements in Free Jumping to Predict Future Show Jumping Performance

To predict the jumping ability of horses in competition from free jumping kinematics, literature very often uses too low a number of horses to calculate genetic correlation and is therefore focused on phenotypic correlation. In several publications (Santamaria et al., 2004a,b, 2005, 2006; Bobbert et al., 2005), the team from Utrecht University managed an experimental design. They measured kinematic data on foals in free jumping and then raised them in two sets with and without training. At 4 years of age, the two groups were joined to start a year long common training program for show jumping. They were kinematically analyzed while free jumping at the beginning of the year and at the end, at the age of 5, they were analyzed during jumping tests under saddle. These tests were all performed on vertical obstacles. The jumping ability of the horses was assessed by a puissance competition under a rider to clear the maximal height. They started with 43 warmblood foals and ended with 29. They showed that certain kinematic variables related to forelimb and hindlimb use, when clearing the obstacle (relative length of the forelimb, elbow angle, stifle angle, and degree of retroflexion of the hind limbs), at foal age had a predictive value for performance of the adult horse. Training at an early age had no effect on ultimate performance. They reported that: “Early training had effaced the differences between potentially good and less good show jumpers in three of three predictive variables and had introduced a false, because not related to performance, difference between the trained and untrained horses in one of them and a nearly significant trend in another. Early training may to a certain extent obscure differences in talent among individuals at the age at which selection events occur.” They concluded with the difficulty to predict “adult jumping capacity (of individual horse) on the basis of kinematic variables collected during submaximal jumps at foal age.” It is difficult to know if the choice of puissance test to qualify the adult jumping capacity influenced this result. This type of event will select the horses with more explosive muscular power but not necessarily horses who can successfully compete in more complex show jumping events with many successive jumps and combinations of obstacles in a short time. The problem they highlighted in the pre-training of horses (Santamaria et al., 2006), also reported by Wejer et al. (2013), could also have occurred in our design and influenced the phenotypic correlation between accelerometric variables and show jumping competition performance. We had no knowledge of how horses were trained before the breeding show. But the genetic correlation between accelerometric variables and show jumping competition performance was calculated mainly with performances of half sibs of horses tested by an accelerometric jumping test. The use of relatives to calculate genetic correlation avoided the confounding of environmental correlation with the genetic correlation, which may occur when calculated from results on both traits on the same horse. Therefore, even if phenotypic correlation may be biased by pre-training, genetic correlation remains correctly calculated. The main difference between their results and the results of our study is that they found a lower height in the center of gravity (CG) over the tested vertical for best horses (Bobbert et al., 2005). We found higher jump duration for best horses, therefore a mechanically higher height of CG during jumping and a longer length jumped. However, another team found similar results to ours. Powers and Harrison (2000) found differences between two groups of good and poor horses (31 horses) for height of CG, which is higher for good horses. Bobbert et al. (2005) provided two reasons why the best horses jumped the lowest height. First, because horses were not required to jump maximally, there was no reason that the CG trajectory would be different between the groups. Second, the optimum trajectory may be a better adjustment to the obstacle than a higher height jumped. The difference to our results may be simply the age of the horse (3 vs. 5 years), the test (free jumping vs. jumping test under saddle for a 5 year old horse), the type of obstacle (oxer vs. vertical) and the way to qualify good and bad horses (competition performances vs. puissance test). Cassiat et al. (2004) found differences in free jumping parameters over 1 m high obstacles between two groups of horses of different competition levels in a national show jumping competition. They analyzed back kinematics, which cannot be easily compared to our jumping test analysis, but they identified some characteristics of differences between good and bad horses (14 horses).

### Comparison of Objective Jump Variables With Judgment Scores

High genetic correlation between judgement scores for free jumping, during stallion or mare tests and show jumping competition performance, have been demonstrated (Thorén Hellsten et al., 2006) and recently confirmed (Ducro et al., 2007a,b; Viklund et al., 2010; Próchniak et al., 2015; Rovere et al., 2017). Calculated genetic correlation between judgement scores for free jumping and results in competitions discussed in this paper agrees with the literature, and is perhaps slightly lower. Lewczuk (2013) was the first to find a correlation between some of the free jumping kinematics variables and the scores of judges. In their study, judgement effect was also significant, meaning that judges had their own definition of a good jump. In our results, judgement scores were also highly genetically correlated with jump duration (DJ), and jump duration (DJ) was highly correlated with performance in a competition. These results are consistent with each other.

The high genetic correlation between judgement scores and results in a competition, and the high genetic correlation between DJ measured by accelerometry and results in competition, allow the use of both traits for jumping horse selection. The advantage of using accelerometric variables rather than judgement scores for horse selection is its objectivity. Judges generally know the pedigree of the horse, or at least recognize the sires, and are aware of their quality for competition. The genetic correlation found between judgement scores and competition are therefore, perhaps, overestimated.

### Reliability of the Results, Improvements of the Method

The strength of the study was the number of horses measured by the same accelerometric device under comparable conditions (1,056). The use of many relatives in a competition, to compute the genetic correlation with accelerometric data, suppressed possible confusion between genetic and environmental correlations. The reliability obtained for the estimated genetic correlation between DJ and jumping performance in a competition was not so high, due to the low number of progeny by stallions, but was sufficient to be significant. Duration of swing phase was also highly heritable in gait analysis according to Sole et al. (2014): 0.37 at trot in hand in Lusitano Purebred horses.

Lewczuk (2017) estimated heritability of distance parameters of jumping from videos with and without riders on a sample of 141 young stallions (3 years). Barrey (2014b) estimated heritability of accelerometric parameters during free jumping of 268 stallions in activity. Both studies had to low a number of horses to be discussed.

Heritability of durations (DFH, DHJ, DJL) other than jump duration (DJ) were very low. It can be explained by the short time between each of these events (between FAT and HAT at takeoff, between HAT and the beginning of a jump, between end of jump and FAL) compared to the duration of the jump. The measured durations were not very accurate, and their standard deviation was too close to the measurement error of the device. Acceleration peaks were heritable and repeatable but were unfortunately not highly correlated to performance in competitions. The most important parameter in free jumping at 3 years old, is perhaps the ability to jump high and far, as measured by DJ, whatever the jumping technique used, measured by the accelerations (FAT, HAT, FAL). The results of this study are not a general observation on jumping technique but only looks at the efficiency of using a free jumping test to select young horses as jumping horses.

Many publications have analyzed the kinematics of horse jumping in more detail than we have done. Their results may inspire new variables to be added to our analysis. Of course, accelerometry cannot measure the positions of the anatomical landmarks on the horse. But perhaps some distances from the horse to the obstacle may be translated in duration from the curve of acceleration. Janczarek et al. (2013) suggested many kinematic variables to describe the jumping process of a horse. The most important seems to be pole—obstacle distance in the last stride before jumping. This variable was negatively correlated to sternum obstacle top distance and jump flight distance, which defined the quality of the jump. They measured a large number of horses (540) during a 100-day stallion test. As in our study, horses were analyzed during a free jumping test and were 3 years old. It would be interesting to further analyze this last stride in our accelerometric data. Another argument for the analysis of the last stride is the work of Bobbert and Santamaria (2005). They calculated the mechanical energy during forelimb and hindlimb push.

The process is being automated (Echterhoff et al., 2018). The studbook plans to continue the recording of young horses during their breeding events. The introduction of such jumping test measurements in the selection plan is being studied and the help of genotypes obtained for the majority of the horses in this study may be the start of a genomic evaluation.

## Conclusion

This study analyzed free jumping during regular breeding shows of young horses with an accelerometric device. For the first time, estimates of genetic correlation between biomechanical data and performance in a competition were calculated. We found that duration of jump was heritable and genetically correlated to jumping performance in a competition. Judgement scores during free jumping were also heritable and correlated to both performance in competition and jump duration measured by the device. The addition of jump duration measured by an accelerometric device in a selection plan must be further studied to replace or complement judgment scores. The advantage of such a measurement, is to be free from the subjectivity of judgement scores, and the calculated correlation cannot be overestimated by *a priori* knowledge of the judges on the quality of the sires of the horses.

## Data Availability Statement

The datasets generated for this study are available on request to the corresponding author.

## Ethics Statement

Ethical review and approval was not required for the animal study because Measurement on horses are part of regular competitions and breeding shows. Written informed consent was obtained from the owners for the participation of their animals in this study.

## Author Contributions

AR performed the statistical analyses, critically interpret the data, and wrote the manuscript. BD supervised recording of data and critically interpret the data. SD managed experimental design. EB made substantial contributions to the interpretation of data and edition of the manuscript.

## Conflict of Interest

The authors declare that the research was conducted in the absence of any commercial or financial relationships that could be construed as a potential conflict of interest.

## Supplementary Material

Supplementary File Variance-Covariance matrices corresponding to Tables 3–7 in the text.
